# Effects of kaempferol on bone loss in animal models of osteoporosis: a systematic review and meta-analysis

**DOI:** 10.3389/fendo.2026.1805337

**Published:** 2026-04-22

**Authors:** Guangming Kang, Dechun Qu, Bo Dong, Rui Tang, Xin Liu, Shihang Cao, Dongping Wan, Haoxiang Yuan, Chuan leng, Rui Wang, Baohui Wang

**Affiliations:** 1 Department of Pain Management, Traditional Chinese Medicine Center, Honghui Hospital, Xi’an Jiaotong University, Xi'an, Shaanxi, China; 2 Medical Department of affiliated traditional Chinese Medicine Hospital of Southwest Medical University, Luzhou, Sichuan, China; 3 The Clinical Medical College, Chengdu University of Chinese Traditional Medicine, Chengdu, Sichuan, China; 4 Zigong Center for Disease Control and Prevention, Zigong, Sichuan, China; 5 The First Clinical Medical College, Guangxi University of Chinese Medicine, Nanning, Guangxi, China

**Keywords:** animal models, bone mineral density, kaempferol, meta-analysis, osteoporosis

## Abstract

**Purpose:**

Osteoporosis remains a major global health challenge, necessitating the search for safe and effective therapeutic leads. Kaempferol, a natural flavonoid, has shown potential in bone health management. This systematic review and meta-analysis aimed to quantitatively evaluate the preclinical efficacy of kaempferol in mitigating bone loss in animal models of osteoporosis.

**Methods:**

Following PRISMA guidelines and PROSPERO registration (CRD420251273304), a comprehensive search was conducted across eight electronic databases up to January 2026. Twelve randomized controlled trials investigating kaempferol monotherapy in osteoporotic animal models (primarily OVX rats) were included. Methodological quality was assessed using SYRCLE’s risk of bias tool, and meta-analysis was performed using Stata 18.0.

**Results:**

Kaempferol significantly increased femoral bone mineral density (BMD) (SMD = 2.86; 95% CI: 1.96–3.79; p < 0.001). It also significantly improved microarchitectural parameters (BV/TV, Tb.N, Tb.Th) and biomechanical properties (elastic modulus). Mechanistically, kaempferol elevated bone formation markers (P1NP), suppressed bone resorption markers (TRACP, CTX), and modulated the RANKL/OPG signaling axis. Subgroup analyses confirmed consistent osteoprotective effects across various dosages and intervention durations.

**Conclusion:**

Preclinical evidence robustly demonstrates that kaempferol effectively preserves bone mass and microarchitectural integrity while enhancing mechanical strength. These findings establish kaempferol as a promising natural bioactive candidate for osteoporosis management and provide a rigorous evidence-based foundation for its clinical translation.

**Systematic Review Registration:**

https://www.crd.york.ac.uk/prospero/, identifier CRD420251273304.

## Introduction

Osteoporosis is a systemic skeletal disorder characterized by reduced bone mass, microarchitectural deterioration, and increased skeletal fragility, culminating in a heightened predisposition to fractures (1, 2). Given the rapidly aging global population, osteoporosis has emerged as a critical public health challenge, imposing a substantial socioeconomic burden and severely compromising patient quality of life and long-term prognosis (3, 4). Fragility fractures associated with osteoporosis are linked to elevated morbidity and mortality rates, representing a primary contributor to chronic disability and extensive healthcare resource utilization among the elderly (5, 6). Pathophysiologically, the progression of osteoporosis is driven by a disruption in bone remodeling, characterized by osteoclast-mediated bone resorption outstripping osteoblast-mediated bone formation (7–9). Estrogen deficiency, particularly in postmenopausal osteoporosis, plays a central role in this process by promoting osteoclast activity, impairing osteoblast function, and amplifying chronic inflammation and oxidative stress, thereby accelerating bone loss and deterioration of skeletal quality (10–12). Although pharmacological agents such as bisphosphonates, hormone replacement therapy, selective estrogen receptor modulators (SERMs), and parathyroid hormone (PTH) analogs are widely employed to mitigate fracture risk (13), their long-term clinical utility is often hampered by adverse effects, safety concerns, and poor patient compliance (14, 15); furthermore, the sustainability of their therapeutic benefits post-discontinuation remains poorly defined. Consequently, there is an urgent need to identify safer and more sustainable intervention strategies for the long-term management of osteoporosis (14, 16). Within this framework, animal models—particularly the ovariectomized (OVX) model—are extensively utilized to recapitulate disease pathogenesis and systematically evaluate the effects of novel therapies on bone mineral density (BMD), microarchitecture, and metabolic biomarkers, thereby providing essential preclinical evidence for clinical translation (17, 18).

Kaempferol, a natural flavonoid ubiquitously distributed in various plant-based foods and traditional Chinese medicines, is prevalent in a wide range of vegetables, fruits, and botanical sources (19, 20). Structurally, it is a flavonol-type flavonoid (3, 4′, 5, 7-tetrahydroxyflavone), and its polyphenolic backbone with multiple hydroxyl groups contributes to its antioxidant and anti-inflammatory properties, which may be beneficial in the prevention of osteoporosis. Accumulating evidence has demonstrated that kaempferol possesses potent anti-inflammatory, antioxidant, and pleiotropic signaling regulatory activities, underscoring its therapeutic potential in cardiovascular (21), metabolic, and inflammation-driven disorders (22). Concomitant with the deepening understanding of bone metabolic regulation, the role of kaempferol in skeletal health has gained increasing prominence (23, 24). Numerous *in vitro* and *in vivo* studies suggest that kaempferol modulates bone remodeling by influencing osteoblast and osteoclast activities, thereby enhancing bone mineral density and preserving microarchitectural integrity (25, 26). Preclinical evidence primarily derived from animal models indicates that kaempferol intervention effectively attenuates bone loss across various experimental paradigms of osteoporosis. However, substantial heterogeneity exists among these studies regarding animal models, dosage regimens, intervention durations, and outcome measures; furthermore, small sample sizes in individual studies often compromise the robustness and comparability of the findings (24, 27, 28). Consequently, a systematic review and meta-analysis are warranted to synthesize and quantitatively analyze the available preclinical data. Such an approach will provide a comprehensive assessment of kaempferol’s overall efficacy in mitigating bone loss and establish a more rigorous evidence-based foundation for its potential clinical translation.

Therefore, the present study was designed to systematically synthesize and quantitatively analyze existing preclinical data concerning the impact of kaempferol on bone loss in various animal models of osteoporosis. The primary objective was to provide a comprehensive evaluation of its overall efficacy on bone mineral density (BMD), microarchitectural parameters, and bone turnover markers. By aggregating data through meta-analysis, we aimed to expand the effective sample size and enhance statistical power while rigorously exploring potential sources of inter-study heterogeneity. This approach seeks to overcome the inherent limitations of individual animal experiments, such as restricted sample sizes and conflicting results. Ultimately, this study intends to establish a more robust preclinical evidence-based foundation for the anti-osteoporotic properties of kaempferol. Furthermore, our findings are expected to offer valuable insights for future mechanistic investigations and facilitate its potential clinical translation.

## Methods

This systematic review and meta-analysis was conducted in strict accordance with the Preferred Reporting Items for Systematic Reviews and Meta-Analyses (PRISMA) guidelines (29). The study protocol has been prospectively registered with the PROSPERO database (registration number: CRD420251273304).

### Search strategy

A comprehensive search was conducted across eight electronic databases, comprising both English (PubMed, Web of Science, Embase, Scopus, and FMRS) and Chinese (CNKI, Wanfang, and VIP) sources. Two reviewers independently performed the literature search to identify relevant studies. The search strategy utilized a combination of Medical Subject Headings (MeSH) terms and free-text keywords. Databases were searched from their inception through January 20, 2026, with an emphasis on studies published between 2000 and 2026 using predefined search strings (detailed in Appendix 1).

### Inclusion and exclusion criteria

This study employed a randomized controlled trial (RCT) design to systematically evaluate and compare the therapeutic efficacy of kaempferol against saline or placebo (vehicle control) in animal models of osteoporosis. Studies were eligible for inclusion if they met the following criteria: (a) Population: validated animal models of osteoporosis; (b) Intervention: kaempferol monotherapy (*in vivo*); (c) Comparison: vehicle or inactive controls; (d) Outcomes: clearly defined parameters with extractable quantitative data; and (e) Study Design: randomized controlled trials. Studies were excluded based on the following: (a) models involving co-morbidities or other metabolic bone disorders; (b) *in vitro* studies, or those involving combination therapies and complex herbal formulae; (c) duplicate publications or overlapping datasets; (d) non-original research, including conference abstracts, reviews, editorials, and letters.

### Data extraction

Following deduplication, two reviewers independently screened titles and abstracts in a blinded manner to exclude studies that failed to meet the predefined eligibility criteria. Subsequently, the full texts of potentially relevant articles were retrieved and scrutinized to confirm their eligibility for inclusion. Any discrepancies during the selection process were resolved through consensus or, if necessary, by consultation with a third reviewer. Data extraction was performed independently by two investigators using a standardized form, adhering to a double-blind protocol. The following variables were extracted: first author, year of publication, method of osteoporosis induction, animal characteristics (species, weight, and age), sample size, dosage and administration route, duration of intervention, and primary outcomes reported as mean and standard deviation (SD). For data presented exclusively in graphical format, numerical values were reconstructed using GetData Graph Digitizer software (Version 2.26).

### Quality assessment

The risk of bias for each included study was independently assessed using the SYRCLE’s risk of bias tool for animal intervention studies (30). This assessment encompassed ten specific items across six core domains: selection bias, performance bias, detection bias, attrition bias, reporting bias, and other potential sources of bias. Each item was categorized as “low risk of bias” if the methodological criteria were satisfied, or “high risk of bias” if the requirements were not met. In instances of inadequate reporting where a definitive judgment could not be made, the risk was designated as “unclear.” Any discrepancies between the two reviewers during the assessment process were resolved via consensual discussion to ensure the objectivity and reliability of the findings.

### Outcome indicators

The primary outcome measure of this study was femoral bone mineral density (BMD). Secondary outcomes included bone histomorphometric parameters, specifically: trabecular number (Tb.N), trabecular thickness (Tb.Th), trabecular separation (Tb.Sp), and bone volume fraction (BV/TV). Biomechanical properties, such as elastic modulus and structure model index (SMI), were also assessed. Bone turnover markers (BTMs) and biochemical indices were evaluated, encompassing procollagen type I N-terminal propeptide (P1NP), alkaline phosphatase (ALP), osteocalcin (OC), tartrate-resistant acid phosphatase (TRACP), C-terminal telopeptide of type I collagen (CTX), as well as serum calcium and phosphorus levels. Furthermore, key regulatory cytokines were analyzed, including osteoprotegerin (OPG), receptor activator of nuclear factor-kappa B ligand (RANKL), and the RANKL/OPG ratio.

### Statistical analysis

Statistical analyses and data visualizations were performed using Stata SE (version 18.0; StataCorp, USA) and Review Manager (version 5.4.0; The Cochrane Collaboration). Inter-study heterogeneity was quantitatively assessed using the I^2^ statistic. A fixed-effects model was employed if I^2^ < 50%, indicating low-to-moderate heterogeneity. Conversely, if I^2^ ≥ 50%, a random-effects model was adopted, unless the source of heterogeneity warranted an alternative approach (31). To further elucidate potential sources of heterogeneity, subgroup and sensitivity analyses were conducted. Sensitivity analysis was performed using the leave-one-out approach to evaluate the stability and robustness of the pooled estimates. For continuous outcomes, therapeutic efficacy was estimated as the standardized mean difference (SMD) with corresponding 95% confidence intervals (CIs). Statistical significance was predefined at a two-tailed p < 0.05.

## Results

### Literature search results

**Figure 1:**
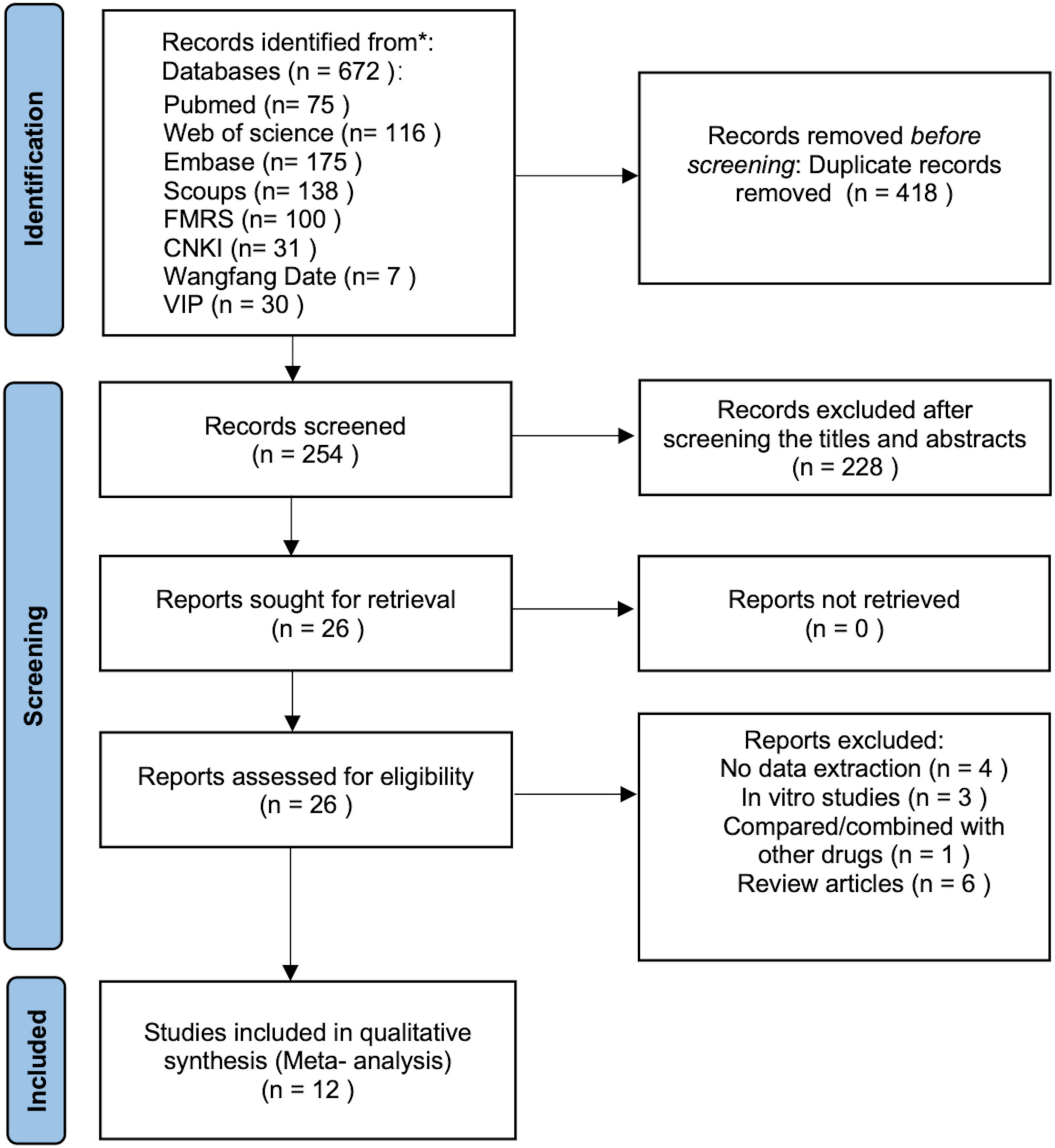
Flowchart of the experimental procedure.

The literature selection process is illustrated in the PRISMA flow diagram (**Figure 1**). A total of 672 records were initially identified across eight electronic databases. Following the removal of 418 duplicates, the titles and abstracts of the remaining 254 articles were screened, resulting in the exclusion of 228 irrelevant studies. The remaining 26 articles were retrieved for full-text evaluation, of which 14 were subsequently excluded. The specific reasons for exclusion were: (a) insufficient data for primary outcomes (n = 4); (b) combination or comparison with other pharmacological agents (n = 1); (c) *in vitro* design (n = 3); and (d) review articles (n = 6). Ultimately, 12 studies (3 in Chinese (32–34) and 9 in English (35–38a, 27, 28, 39–41)) met all eligibility criteria and were included in this meta-analysis.

### Characteristics of included studies

The fundamental characteristics of the included studies are summarized in **Table 1**. The 12 identified studies were published between 2008 and 2025. Regarding the experimental models, eleven studies utilized ovariectomy (OVX) to induce osteoporosis, while one study employed glucocorticoid-induced osteoporosis. All experiments were conducted using rat models. For the control groups, the administered vehicles consisted of normal saline (n = 6), 0.5% CMC-Na (n = 5), and distilled water (n = 1). Both intervention and control groups received treatments via oral gavage. The dosages of kaempferol ranged from 1 to 50 mg/kg/day, with intervention durations spanning from 4 to 12 weeks.

**Table 1:** Characteristics of the included studies.

First author	Induction of osteoporosis	Species	Gender	Sample size		Intervention		Methods of administration	Duration of study
				IG	CG	IG	CG		
(34)	OVX	SD	Female	8	8	50 mg/(kg·d)	CMC-Na	Intragastric	12 weeks
(25)	OVX	SD	Female	12	12	40 mg/(kg·d)	PS	Intragastric	12 weeks
(32)	OVX	SD	Female	8	8	40 mg/(kg·d)	PS	Intragastric	12 weeks
(35)	GIOP	SD	Female	10	10	5 mg/(kg·d)	DW	Intragastric	4 weeks
(39)	OVX	SD	Female	8	8	16 mg/(kg·d)	CMC-Na	Intragastric	12 weeks
(28)	OVX	SD	Female	10	10	5 mg/(kg·d)	PS	Intragastric	10 weeks
(27)	OVX	Wistar	Female	8	8	5 mg/(kg·d)	PS	Intragastric	8 weeks
(36)	OVX	SD	Female	12	12	1 mg/(kg·d)	PS	Intragastric	4 weeks
(41)	OVX	SD	Female	6	6	10 mg/(kg·d)	CMC-Na	Intragastric	10 weeks
(40)	OVX	SD	Female	10	10	40 mg/(kg·d)	CMC-Na	Intragastric	12 weeks
(37)	OVX	SD	Female	12	12	5 mg/(kg·d)	CMC-Na	Intragastric	12 weeks
(38)	OVX	SD	Female	8	8	20mg/(kg·d)	PS	Intragastric	12 weeks

OVX, Ovariectomy; GIOP, Glucocorticoid-induced Osteoporosis; IG, Intervention Group; CG, Control Group; PS, physiological saline; CMC-Na, Sodium Carboxymethylcellulose; DW, Distilled water.

### Methodological quality of included studies

**Figure 2:**
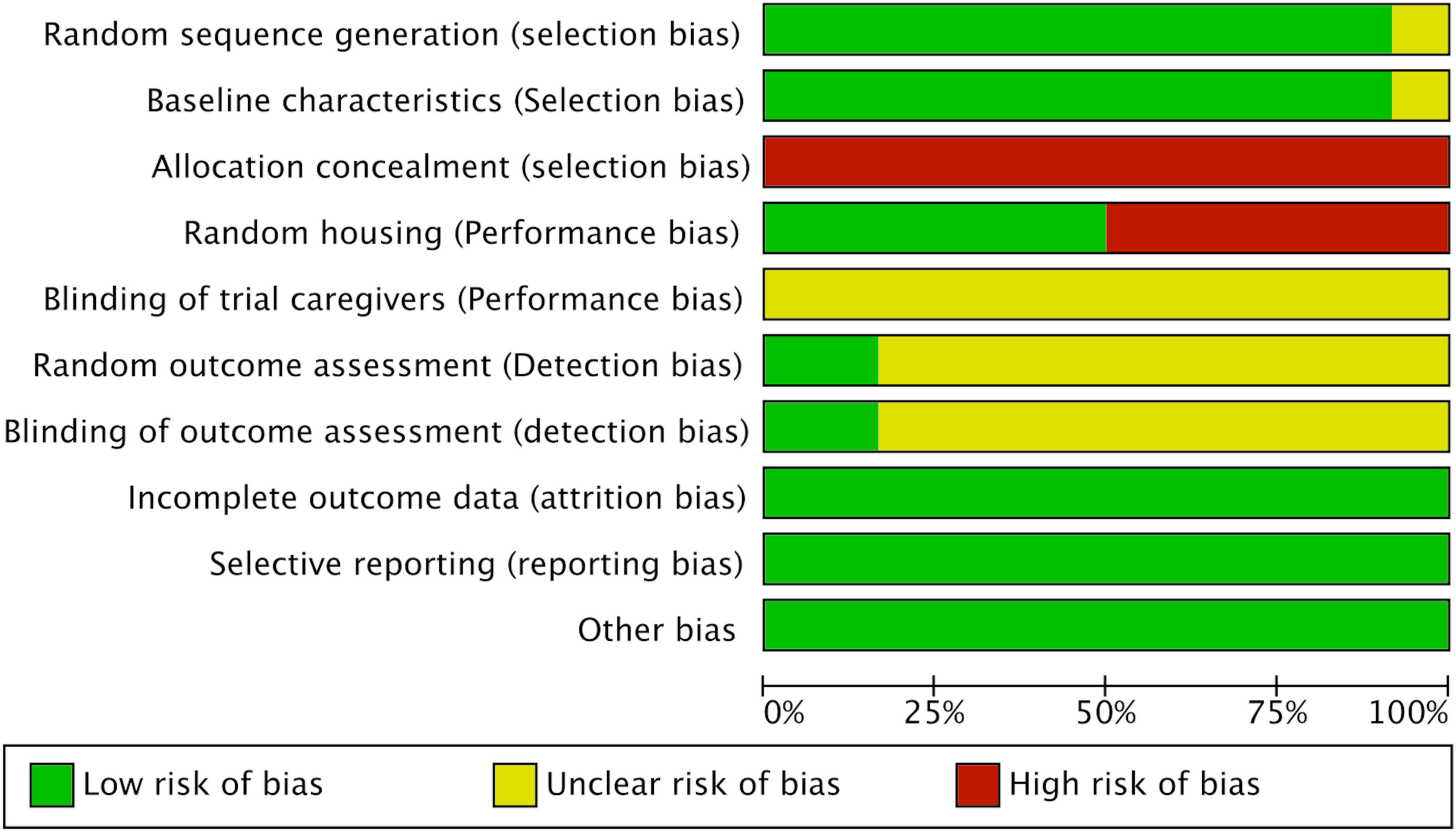
Quality assessment of the included studies.

The methodological quality of the 12 included studies was independently assessed using the SYRCLE’s risk of bias tool (**Figure 2**). Overall, the included studies demonstrated a moderate-to-high level of methodological quality. Regarding selection bias, 11 studies reported random sequence generation, while one failed to provide a description; baseline characteristics were balanced across groups as previously detailed. Notably, allocation concealment was not explicitly addressed in any of the identified studies. Concerning performance bias, six studies implemented random housing for the experimental animals. However, given the practical constraints inherent in animal research, only a limited number of studies reported blinding of personnel. For detection bias, the application of random outcome assessment and investigator blinding remained poorly documented. Conversely, all studies were deemed to have a low risk of attrition and reporting biases. The datasets were complete, and no evidence of selective outcome reporting was identified.

### Bone mineral density and subgroup analysis

**Figure 3:**
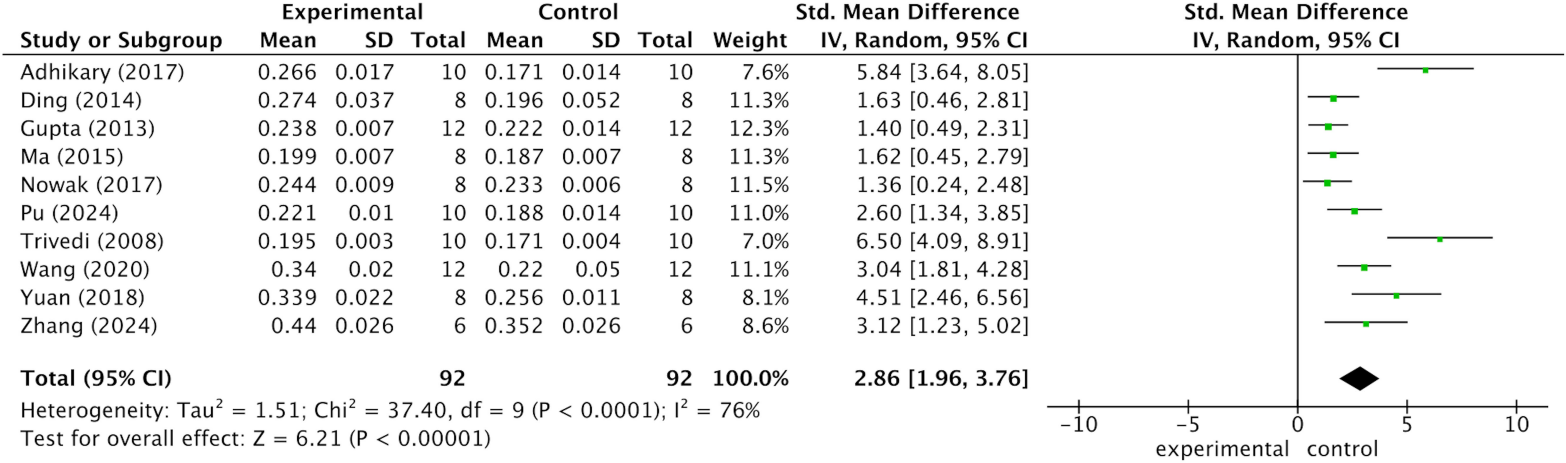
Bone mineral density (BMD) of the femur.

Data synthesis from 10 studies corroborated the efficacy of kaempferol in attenuating bone mineral density (BMD) loss in osteoporotic animal models. As illustrated in **Figure 3**, femoral BMD was significantly higher in the kaempferol-treated group compared to the control group (SMD = 2.86; 95% CI: 1.96–3.79; p < 0.001). To elucidate potential sources of heterogeneity and evaluate the robustness of kaempferol’s effects across diverse experimental conditions, a stratified subgroup analysis was performed (summarized in **Table 2**). Notably, kaempferol demonstrated consistent and significant osteoprotective effects across all predefined subgroups (p < 0.001). Within the ovariectomized (OVX) model subset, the pooled effect size (SMD = 2.56) confirmed that kaempferol effectively mitigated bone loss secondary to estrogen deficiency. Interestingly, the effect size in the low-dose group (<10 mg/kg/d; SMD = 3.40) was numerically greater than that of the high-dose group (>10 mg/kg/d; SMD = 2.49), albeit with substantial heterogeneity (I^2^ = 86%). Regarding the duration of intervention, short-term treatment (<12 weeks; SMD = 3.40) appeared more efficacious than long-term treatment (>12 weeks; SMD = 2.49); however, the latter exhibited markedly lower heterogeneity (I^2^ = 53%). Subgrouping by vehicle type revealed that studies utilizing CMC-Na (I^2^ = 52%) showed improved consistency compared to those employing physiological saline (I^2^ = 79%). Additionally, studies with larger sample sizes (n > 8) yielded a higher effect size (SMD = 3.60) than those with smaller cohorts (SMD = 2.18).

**Table 2:** Subgroup analysis of BMD based on multiple factors related to kaempferol treatment.

Subgroup	Standardized mean difference (95% confidence interval)	I^2^	p value
Model			
OVX	2.56 [1.73, 3.39]	71	0.000
Species			
SD Rat	3.07 [2.08, 4.07]	77	0.000
Control Group			
PS	2.45 [1.22, 3.68]	79	0.000
CMC-Na	2.75 [1.64, 3.85]	52	0.000
Dose			
≤ 10 mg/(kg·d)	3.40 [1.57, 5.23]	86	0.000
> 10 mg/(kg·d)	2.49 [1.62, 3.36]	53	0.000
Sample Size			
≤ 8	2.18 [1.23, 3.12]	56	0.000
> 8	3.60 [1.98, 5.22]	84	0.000
Duration of intervention			
< 12 weeks	3.40 [1.57, 5.23]	86	0.003
≥ 12weeks	2.49 [1.62, 3.36]	53	0.000

### Bone histomorphometry

**Figure 4:**
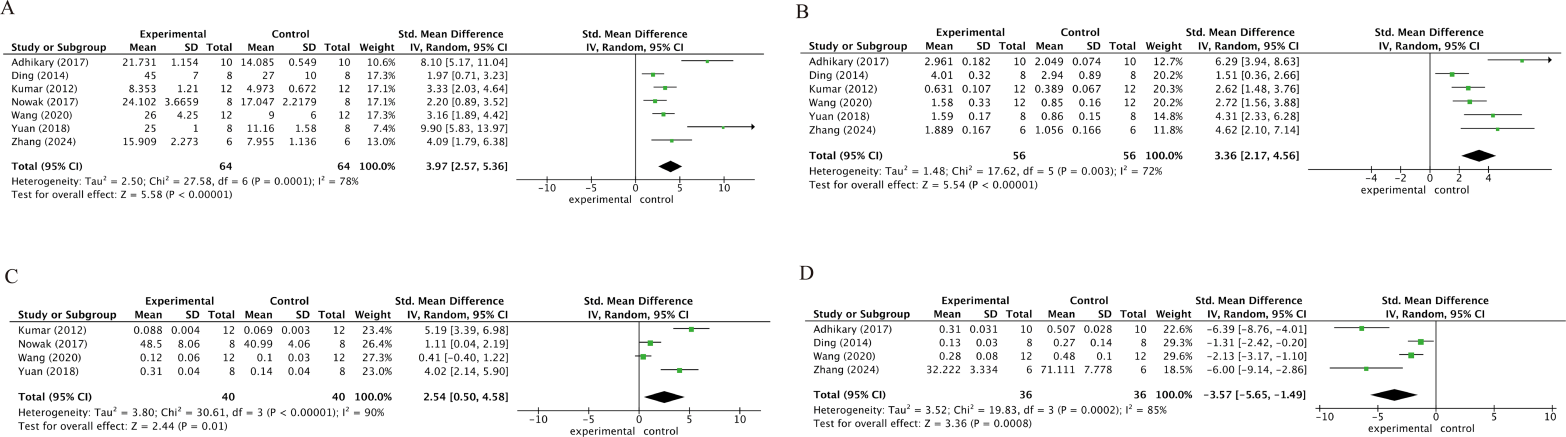
Micro-CT analysis of trabecular bone microarchitecture: **(a)** bone volume fraction (BV/TV); **(b)** trabecular number (Tb.N); **(c)** trabecular thickness (Tb.Th); **(d)** trabecular separation (Tb.Sp).

**Figure 4** illustrates the impact of kaempferol on bone histomorphometric parameters in osteoporotic animal models. Specifically, a meta-analysis of seven studies revealed a significant increase in bone volume fraction (BV/TV; SMD = 3.97; 95% CI: 2.57–5.36; p < 0.001, **Figure 4A**). Similarly, data from six studies demonstrated that kaempferol intervention significantly augmented trabecular number (Tb.N; SMD = 3.36; 95% CI: 2.17–4.56; p < 0.001, **Figure 4B**). Furthermore, four datasets indicated a substantial enhancement in trabecular thickness (Tb.Th; SMD = 2.54; 95% CI: 0.50–4.58; p = 0.01, **Figure 4C**). Concurrently, four studies reported that kaempferol effectively diminished trabecular separation (Tb.Sp; SMD = -3.57; 95% CI: -5.65 to -1.49; p < 0.001, **Figure 4D**).

### Bone biomechanical parameters

**Figure 5:**
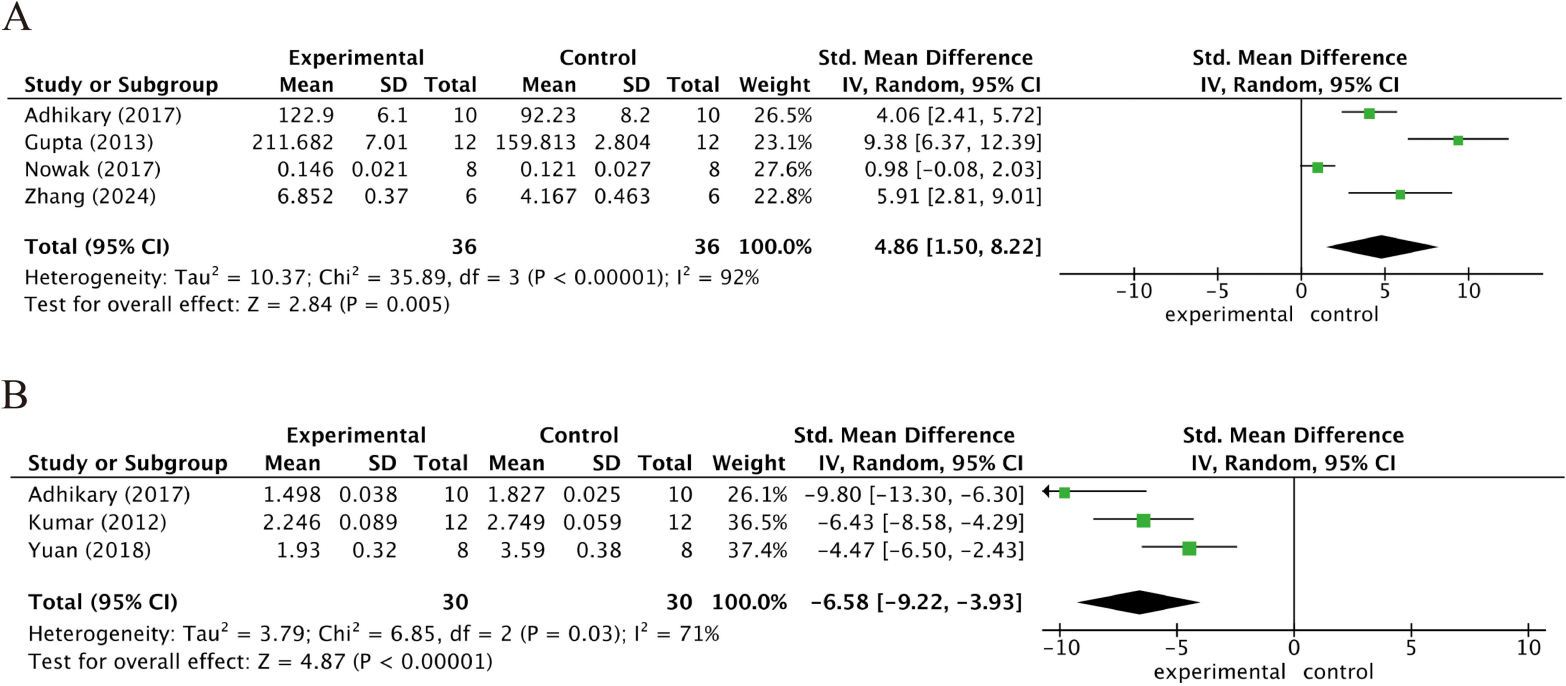
Biomechanical and structural parameters: **(a)** elastic modulus; **(b)** structural model index (SMI).

**Figure 5** summarizes the impact of kaempferol intervention on the biomechanical properties of bone in osteoporotic animal models. Specifically, a synthesis of four studies indicated that kaempferol treatment significantly enhanced the elastic modulus (SMD = 4.86; 95% CI: 1.50–8.22; p = 0.005, **Figure 5A**). Additionally, three studies reported a marked reduction in the structure model index (SMI) following kaempferol administration (SMD = -6.58; 95% CI: -9.22 to -3.93; p < 0.001, **Figure 5B**), reflecting a transition toward a more favorable trabecular architecture.

### Bone turnover markers

**Figure 6:**
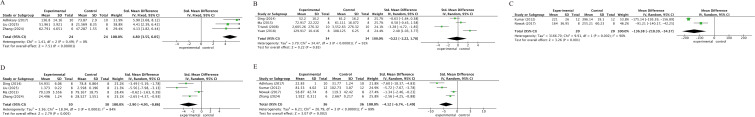
Serum bone turnover markers: **(a)** P1NP; **(b)** ALP; **(c)** OC; **(d)** TRACP; **(e)** CTX.

**Figure 6** summarizes the meta-analysis results regarding the effects of kaempferol on bone turnover markers (BTMs) in osteoporotic animal models. Regarding bone formation markers (**Figures 6A–C**), data from three studies indicated a significant elevation in procollagen type I N-terminal propeptide (P1NP) levels following kaempferol treatment (SMD = 4.80; 95% CI: 3.55–6.05; p < 0.001, **Figure 6A**). Conversely, a synthesis of four studies suggested that kaempferol had no significant impact on alkaline phosphatase (ALP) levels (SMD = -0.22; 95% CI: -2.22 to 1.78; p = 0.83, **Figure 6B**). Additionally, two studies reported a significant reduction in osteocalcin (OC) levels in the kaempferol-treated group (MD = -136.38; 95% CI: -218.39 to -54.37; p = 0.001, **Figure 6C**). In terms of bone resorption markers (**Figures 6D, E**), kaempferol intervention led to a marked decline in tartrate-resistant acid phosphatase (TRACP) levels across four studies (SMD = -2.90; 95% CI: -4.95 to -0.86; p = 0.005, **Figure 6D**). Similarly, four studies documented a significant attenuation of C-terminal telopeptide of type I collagen (CTX) levels (SMD = -4.12; 95% CI: -6.74 to -1.49; p = 0.002, **Figure 6E**).

### Cytokines and mineral homeostasis

**Figure 7:**
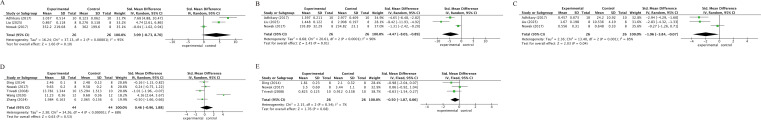
Biochemical indicators and signaling factors: **(a)** OPG; **(b)** RANKL; **(c)** RANKL/OPG ratio; **(d)** S-Ca (serum calcium); **(e)** S-P (serum phosphorus).

**Figure 7** presents the meta-analytical synthesis of kaempferol’s effects on bone signaling pathways and systemic mineral homeostasis in osteoporotic models. Regarding the RANKL/OPG axis (**Figures 7A–C**), a synthesis of three studies indicated a significant elevation in osteoprotegerin (OPG) levels (SMD = 3.99; 95% CI: -0.73 to 8.70; p = 0.01, **Figure 7A**). Concurrently, kaempferol treatment led to a marked downregulation of receptor activator of nuclear factor-kappa B ligand (RANKL) (SMD = -4.47; 95% CI: -8.05 to -0.89; p = 0.01, **Figure 7B**). Consequently, the RANKL/OPG ratio was significantly diminished (SMD = -1.96; 95% CI: -3.84 to -0.07; p = 0.04, **Figure 7C**), suggesting a suppressed osteoclastogenic drive. Indicators of systemic mineral homeostasis were analyzed in **Figures 7D, E**. Pooled data from five studies showed that serum calcium (S-Ca) levels remained unaffected by the intervention (SMD = 0.46; 95% CI: -0.96 to 1.88; p = 0.53). Similarly, no significant alterations were observed in serum phosphorus (S-P) levels across three studies (SMD = -0.50; 95% CI: -1.07 to 0.06; p = 0.08).

### Sensitivity analysis

**Figure 8:**
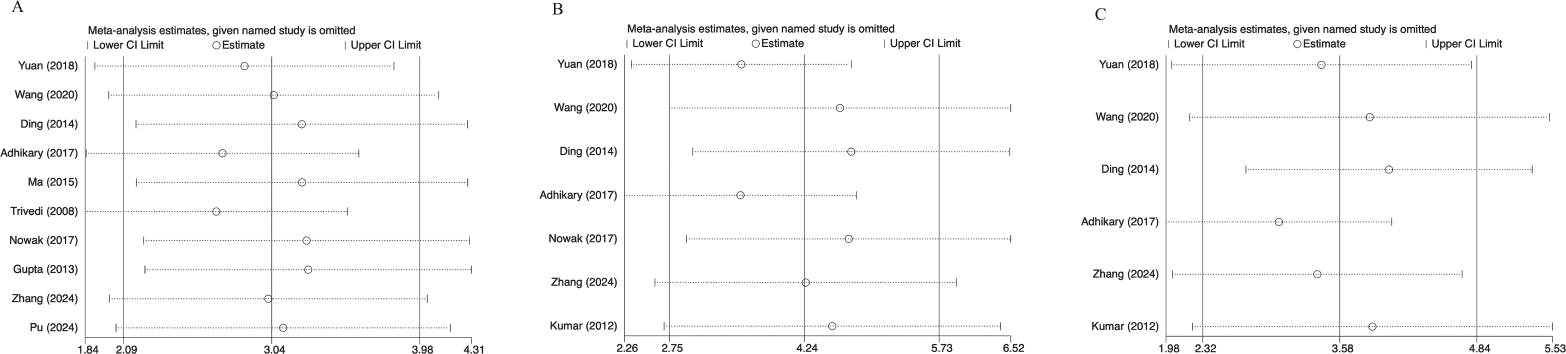
Sensitivity analysis for: **(a)** bone mineral density (BMD); **(b)** bone volume fraction (BV/TV); **(c)** trabecular number (Tb.N).

To evaluate the stability and reliability of our findings, a leave-one-out sensitivity analysis was performed on the pooled estimates of key parameters, including BMD, BV/TV, and Tb.N. Sensitivity analysis for BMD (**Figure 8A**) demonstrated that the pooled standardized mean difference (SMD) remained remarkably stable, with the recalculated estimates following the sequential exclusion of individual studies fluctuating narrowly around the overall effect line (SMD = 3.04). No individual study exerted a disproportionate influence on the final outcome, underscoring the robustness of the BMD meta-analysis. As illustrated in **Figure 8B**, the pooled effect size for BV/TV was not materially altered by any single dataset. The combined SMD and its corresponding 95% CI remained consistently to the right of the null line, confirming the statistical reliability of kaempferol’s bone-protective effects. Similarly, the Tb.N results exhibited substantial stability (**Figure 8C**); although the exclusion of specific studies (e.g., 35 or 32) caused minor numerical shifts, the overall trend and statistical significance remained unchanged. Collectively, these sensitivity analyses indicate that the primary findings of this meta-analysis are stable and not contingent upon any single study with an outlying influence.

## Discussion

This systematic review and meta-analysis provides a comprehensive synthesis of current preclinical evidence regarding the interventional efficacy of kaempferol against bone loss in various osteoporosis models. Our primary findings demonstrate that kaempferol exerts significant osteoprotective effects across diverse experimental paradigms of osteoporosis. Beyond effectively augmenting femoral bone mineral density (BMD), kaempferol treatment resulted in consistent improvements across multiple dimensions, including bone histomorphometry, biomechanical competence, and metabolic biomarkers. Collectively, these data bolster the therapeutic promise of kaempferol in mitigating skeletal deterioration within preclinical settings. Furthermore, this study establishes a rigorous, evidence-based foundation to support future mechanistic investigations and the potential clinical translation of kaempferol.

Regarding bone mass and microarchitecture, our meta-analysis revealed that kaempferol intervention significantly augmented bone mineral density (BMD). This was coupled with concurrent improvements in bone volume fraction (BV/TV), trabecular number (Tb.N), and trabecular thickness (Tb.Th), alongside a significant reduction in trabecular separation (Tb.Sp). Notably, while elevated BMD is widely recognized as a pivotal clinical endpoint in anti-osteoporotic therapy, alterations in BMD alone may not fully encapsulate the overall enhancement of bone quality. In the present study, multiple parameters reflecting microarchitectural integrity exhibited concordant and substantial improvements. (42, 43) These findings indicate that kaempferol’s osteoprotective effects extend beyond increases in bone mass, as it improves the microarchitectural integrity of trabecular bone by enhancing bone formation signaling and suppressing osteoclastogenic pathways, resulting in improved connectivity and reduced trabecular separation (38b). Such comprehensive microarchitectural restoration has critical implications for mitigating skeletal fragility and fracture risk, further reinforcing the biological plausibility of our findings.

Furthermore, the enhancement of bone biomechanical parameters provides crucial functional validation for the aforementioned structural improvements. Our findings demonstrate that kaempferol significantly increases the elastic modulus and reduces the structural model index (SMI). This suggests a substantial fortification of the bone tissue in terms of both load-bearing capacity and overall mechanical competence. Improvements in biomechanical properties reflect the functional integrity of bone tissue under physiological loading conditions. Compared to static morphological or radiographic markers, these parameters offer a more direct and comprehensive assessment of bone quality. (44, 45) The highly convergent trends observed across structural, mechanical, and bone mass indicators in this study underscore the robustness of our data. These findings indicate that the interventional effects of kaempferol exhibit strong internal consistency and biological reliability within the osteoporotic model.

Regarding bone turnover markers (BTMs), the meta-analysis results revealed that kaempferol intervention significantly elevated the levels of procollagen type I N-terminal propeptide (P1NP), a key indicator of bone formation. Concurrently, there was a marked reduction in bone resorption markers, specifically TRACP and CTX. However, ALP and osteocalcin (OC) showed variable responses across studies, which may be due to differences in experimental protocols, sampling time points, and kaempferol dosages. Despite this variability, the consistent trend of kaempferol’s ability to increase bone formation and reduce bone resorption suggests that kaempferol effectively modulates skeletal metabolism in a favorable manner. (46) Nevertheless, the predominant trend underscores the potential of kaempferol to modulate skeletal metabolism in a favorable manner.

Furthermore, at the molecular level, our analysis revealed that kaempferol intervention significantly elevated OPG levels while concomitantly reducing RANKL expression and the RANKL/OPG ratio. The RANKL/OPG signaling axis represents a pivotal regulatory pathway for osteoclast differentiation and activation; its dysregulation is widely recognized as a central mechanism in the pathogenesis of osteoporosis. (47–49) The observed alterations in these markers are congruent with the findings of attenuated bone resorption. This suggests that the osteoprotective efficacy of kaempferol may be mediated, at least in part, by modulating the RANKL/OPG axis to suppress osteoclast-mediated bone resorption. *In vitro* studies have shown that kaempferol inhibits osteoclast differentiation by downregulating RANKL and related signaling pathways such as c-Fos and NFATc1 (50). *In vivo* studies, including those conducted in OVX rats, have demonstrated that kaempferol increases OPG expression and suppresses RANKL expression, leading to reduced bone resorption and improved bone mass (38). It should be noted that, as a meta-analysis of preclinical studies, our findings primarily reflect mechanistic correlations rather than definitive causality. Nevertheless, these results demonstrate high concordance with previously reported *in vivo* and *in vitro* evidence.

**Figure 9:**
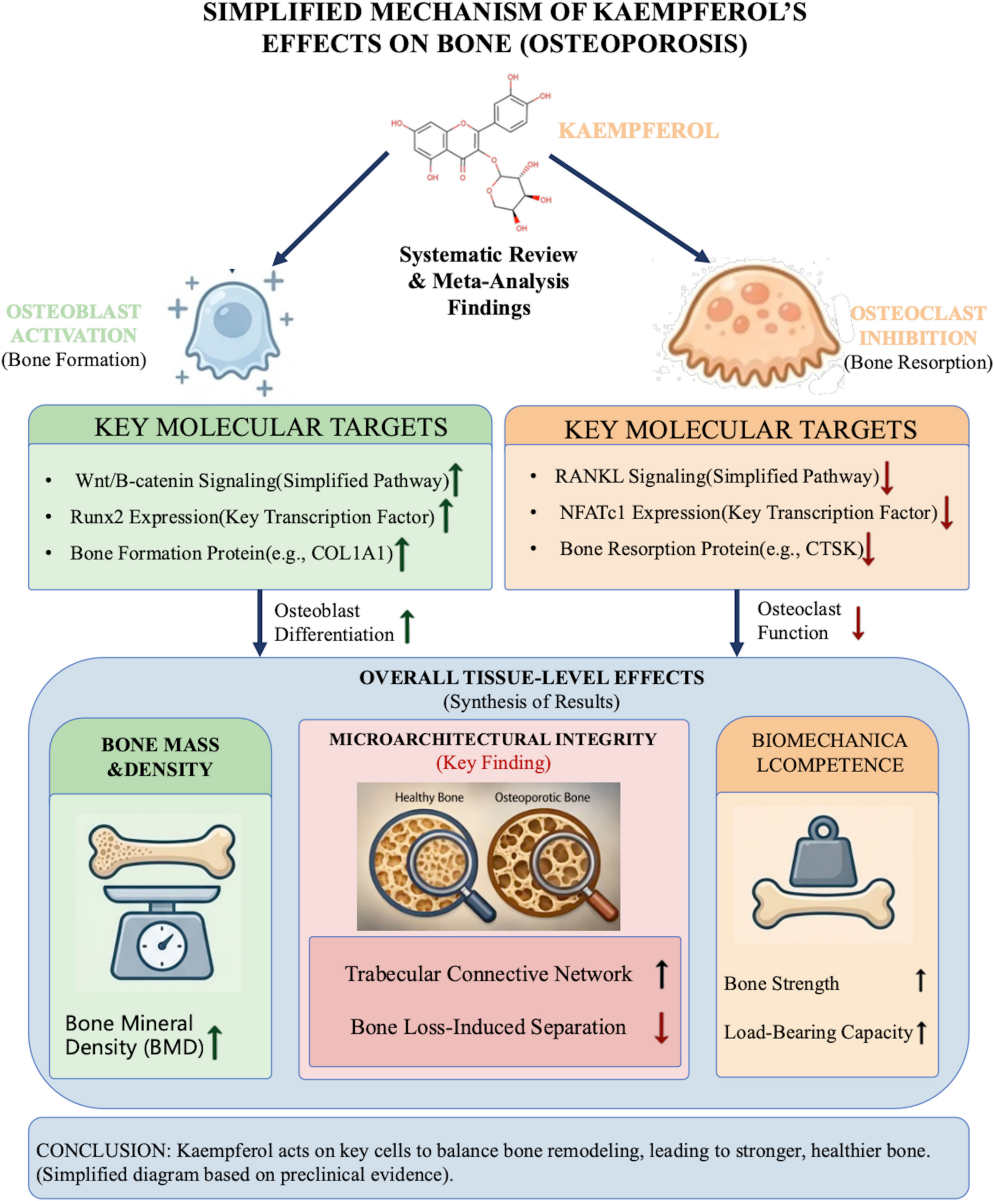
Simplified schematic summary of the proposed anti-osteoporotic mechanisms of kaempferol.

The results of our subgroup analyses provide further insights into the inter-study heterogeneity and the pharmacological profile of kaempferol. Kaempferol consistently demonstrated significant osteoprotective effects across various animal models, dosages, intervention durations, and vehicle types, underscoring its therapeutic robustness. Notably, the low-dose group (<10 mg/kg/d) exhibited a more pronounced effect magnitude numerically, albeit accompanied by heightened heterogeneity. This variability may be attributed to discrepancies in experimental design, limited sample sizes, or the potentially non-linear dynamics of the dose-response relationship. Furthermore, studies characterized by extended intervention periods and larger cohorts typically manifested diminished heterogeneity. This suggests that the methodological standardization and statistical power of a study are critical determinants of the stability of effect size estimates. Collectively, these findings offer valuable benchmarks for optimizing dosage selection and experimental protocols in future preclinical investigations. To facilitate interpretation of the overall findings, **Figure 9** provides a simplified schematic summary of the proposed mechanisms by which kaempferol exerts anti-osteoporotic effects, including the promotion of osteoblast activity, inhibition of osteoclastogenesis, and the consequent improvement in bone mass, microarchitectural integrity, and biomechanical competence.

Despite the comprehensive synthesis of the available evidence, several limitations of the current study should be acknowledged. Primarily, the included studies relied heavily on the ovariectomized (OVX) rat model; while this paradigm is a gold standard for mimicking postmenopausal osteoporosis, the extrapolation of these preclinical findings to human clinical settings should be interpreted with caution. (51) Furthermore, a subset of the analyzed studies exhibited suboptimal methodological transparency regarding randomization, blinding, and allocation concealment, which may introduce potential bias into the pooled estimates. (52, 53) Moreover, the inherent heterogeneity arising from variations in dosage regimens, intervention durations, and outcome assessment methodologies across studies may affect the precision of the summarized effect sizes. (54) Finally, given the relatively small number of eligible studies, the potential influence of publication bias cannot be entirely discounted.

In conclusion, this systematic review and meta-analysis demonstrates that kaempferol exerts significant beneficial effects on bone mass, microarchitectural integrity, biomechanical competence, and bone turnover markers in animal models of osteoporosis. Its osteoprotective efficacy remains remarkably robust across a diverse range of experimental conditions. These findings establish a systematic preclinical evidence base supporting kaempferol as a promising natural bioactive compound for the management of osteoporosis. However, additional pharmacokinetic, toxicological, and clinical studies are necessary to fully evaluate its therapeutic potential and safety before progressing toward clinical application.

## Data Availability

The original contributions presented in the study are included in the article/**Supplementary Material**. Further inquiries can be directed to the corresponding author.
