# Supplementary material for: Effects of kaempferol on bone loss in animal models of osteoporosis: a systematic review and meta-analysis
Source: Front Endocrinol (Lausanne). 2026 Apr 22;17:1805337. doi: 10.3389/fendo.2026.1805337 (PMC13143670; doi:10.3389/fendo.2026.1805337)
Supplement: Supplementary file 1 [file DataSheet1.docx]

**Pubmed**

((("Kaempferol"[MeSH] OR kaempferol[tiab] OR "KMP"[tiab])) AND (("Osteoporosis"[MeSH] OR osteoporosis[tiab] OR "bone loss"[tiab] OR "bone mineral density"[tiab] OR BMD[tiab]))) AND ((animal[tiab] OR animals[tiab] OR rat[tiab] OR rats[tiab] OR mice[tiab] OR mouse[tiab] OR ovariectomiz*[tiab] OR "cell line"[tiab] OR osteoblast*[tiab] OR osteoclast*[tiab] OR "in vitro"[tiab] OR "in vivo"[tiab]))

**Web of science**

TS=((("Kaempferol" OR kaempferol OR "KMP") AND ("Osteoporosis" OR osteoporosis OR "bone loss" OR "bone mineral density" OR BMD)) AND ((animal OR animals OR rat OR rats OR mice OR mouse OR ovariectomiz* OR "cell line" OR osteoblast* OR osteoclast* OR "in vitro" OR "in vivo")))

**Embase**

(('kaempferol':de OR 'kaempferol':ti,ab OR 'kmp':ti,ab) AND ('osteoporosis':de OR 'osteoporosis':ti,ab OR 'bone loss':ti,ab OR 'bone mineral density':ti,ab OR 'bmd':ti,ab) AND ('animal':ti,ab OR 'animals':ti,ab OR 'rat':ti,ab OR 'rats':ti,ab OR 'mice':ti,ab OR 'mouse':ti,ab OR ovariectomiz*:ti,ab OR 'cell line':ti,ab OR osteoblast*:ti,ab OR osteoclast*:ti,ab OR 'in vitro':ti,ab OR 'in vivo':ti,ab))

**FMRS**

(M=kaempferol OR T=kaempferol OR A=kaempferol OR T=kmp OR A=kmp) AND (M=osteoporosis OR T=osteoporosis OR A=osteoporosis OR T="bone loss" OR A="bone loss" OR T="bone mineral density" OR A="bone mineral density" OR T=bmd OR A=bmd) AND (T=animal OR A=animal OR T=animals OR A=animals OR T=rat OR A=rat OR T=rats OR A=rats OR T=mice OR A=mice OR T=mouse OR A=mouse OR T=ovariectomiz* OR A=ovariectomiz* OR T="cell line" OR A="cell line" OR T=osteoblast* OR A=osteoblast* OR T=osteoclast* OR A=osteoclast* OR T="in vitro" OR A="in vitro" OR T="in vivo" OR A="in vivo")

**Scoups**

TITLE-ABS-KEY((kaempferol OR kaempherol OR kampferol OR kampherol OR kaemferol OR "3,4',5,7-tetrahydroxyflavone" OR "3,5,7-trihydroxy-2-(4-hydroxyphenyl)-4H-chromen-4-one" OR robigenin OR rhamnolutein OR trifolitin OR swartziol OR "indigo yellow" OR pelargidenolon OR nimbecetin OR NSC-656277 OR NSC-407289 OR kampcetin OR "3,5,7,4'-tetrahydroxyflavone") AND (osteoporosis OR "bone loss" OR "bone mineral density" OR "bone mass" OR "bone strength" OR BMD OR BMC OR "bone density" OR "trabecular bone" OR "cortical bone") AND (rat OR rats OR mouse OR mice OR murine OR rodent OR rodents OR "experimental animal*" OR "animal model*" OR "laboratory animal*"))
